# Prehospital identification of factors associated with death during one‐year follow‐up after acute stroke

**DOI:** 10.1002/brb3.987

**Published:** 2018-05-16

**Authors:** Per‐Olof Hansson, Magnus Andersson Hagiwara, Peter Brink, Johan Herlitz, Birgitta Wireklint Sundström

**Affiliations:** ^1^ Department of Molecular and Clinical Medicine Institute of Medicine Sahlgrenska Academy University of Gothenburg Gothenburg Sweden; ^2^ Faculty of Caring Science, Work Life and Social Welfare Centre for Prehospital Research University of Borås Borås Sweden; ^3^ Department of Health Sciences Section for nursing – undergraduate level University West Trollhättan Sweden

**Keywords:** acute stroke, early chain, mortality, one‐year follow‐up

## Abstract

**Objectives:**

In acute stroke, the risk of death and neurological sequelae are obvious threats. The aim of the study was to evaluate the association between various clinical factors identified by the emergency medical service (EMS) system before arriving at hospital and the risk of death during the subsequent year among patients with a confirmed stroke.

**Material and Methods:**

All patients with a diagnosis of stroke as the primary diagnosis admitted to a hospital in western Sweden (1.6 million inhabitants) during a four‐month period were included. There were no exclusion criteria.

**Results:**

In all, 1,028 patients with a confirmed diagnosis of stroke who used the EMS were included in the analyses. Among these patients, 360 (35%) died during the following year. Factors that were independently associated with an increased risk of death were as follows: (1) high age, per year OR 1.07; 95% CI 1.05‐1.09; (2) a history of heart failure, OR 2.08; 95% CI 1.26‐3.42; (3) an oxygen saturation of <90%, OR 8.05; 95% CI 3.33‐22.64; and (4) a decreased level of consciousness, OR 2.19; 95% CI 1.61‐3.03.

**Conclusions:**

Among patients with a stroke, four factors identified before arrival at hospital were associated with a risk of death during the following year. They were reflected in the patients’ age, previous clinical history, respiratory function, and the function of the central nervous system.

## 1 INTRODUCTION

Stroke is a severe manifestation of cardiovascular disease and it is one of the leading causes of morbidity and mortality worldwide (Lopez, Mathers, Ezzati, Jamison, & Murray, 2006). The early phase after the onset of symptoms in acute stroke is particularly critical.

Several previous studies have shown that there is a prehospital delay in acute stroke care due to failure to recognize the symptoms of stroke (Le Bonniec et al., 2016) and only 50%–75% of acute stroke patients use the emergency medicine services (EMS) for transportation to hospital (Andersson Hagiwara, Wireklint Sundström, Brink, Herlitz, & Hansson, 2018; Centers for Disease Control and Prevention (CDC), 2007; Wireklint Sundström, Herlitz, Hansson, & Brink, 2015), despite large educational programmes (Lecouturier et al., 2010). The health‐care providers (HCP) at the dispatch center and the EMS clinicians are often the first HCPs that communicate with patients or bystanders and it is of the utmost importance that patients suffering an acute stroke are rapidly identified before arriving at hospital (Wireklint Sundström et al., 2015). They can then be quickly triaged to emergency clinical pathways and hopefully be offered the optimal treatment at an early stage (Centers for Disease Control and Prevention (CDC), 2007; Lees et al., 2010). However, despite this knowledge, there is still a considerable delay in acute stroke care (Centers for Disease Control and Prevention (CDC), 2007; Evenson, Foraker, Morris, & Rosamond, 2009; Wireklint Sundström et al., 2015).

There are many ways to classify stroke symptoms and severity and the most widely used by HCPs is the National Institutes of Health Stroke Scale (NIHSS) (Goldstein & Samsa, 1997).

The recognition of an acute stroke and the priority given at the dispatch center are related to risk of death (Andersson Hagiwara et al., 2018). However, with regard to the risk of death, factors other than the stroke itself (such as age and comorbidity) may influence the accuracy of this kind of prediction.

The aim of this study was to evaluate the association between various clinical factors and the risk of death during the following year among patients with a confirmed stroke after being brought to hospital by the EMS.

## 2 MATERIAL AND METHODS

The study has an observational, retrospective design and was conducted as a multicenter study. The data were collected during a period of 4 months from 15 December 2010 to 15 April 2011. The study was approved by the Regional Ethical Review Board in Gothenburg (reference number: 514‐10). The reporting of this study conforms with the Strengthening of Reporting of Observational Studies in Epidemiology (STROBE) statement (STROBE, 2007). The study deign has previously been described in detail (Andersson Hagiwara et al., 2018; Wireklint Sundström et al., 2015).

### 2.1 Setting

The study setting was the EMS and the dispatch center in western Sweden. The western region of Sweden has approximately 1.6 million inhabitants and nine emergency hospitals, with a stroke unit at each hospital. Treatment with thrombolysis was available in seven of the nine hospitals, whereas treatment with thrombectomy was only available at one hospital (Sahlgrenska University Hospital). In the EMS, there were 84 ambulances during the study period, all staffed by two EMS nurses or one EMS nurse and one emergency medical technician day and night every day of the week. The EMS nurses were at least registered nurses (RN) and some of them also had specialist education in prehospital emergency care.

### 2.2 Study sample

Patients were included in the study:

1. If they were admitted to a hospital ward and had received acute stroke as the primary diagnosis at discharge (intracerebral hemorrhage, unspecific brain hemorrhage, cerebral infarction, or stroke not classified as infarction or hemorrhage (International Classification of Diseases 10th Revision (ICD‐10): I61.0‐I64.9, with the exception of I62.0 and I62.1.
2. If they were transported by the EMS.

Patients with a diagnosis of stroke were excluded from the study if symptom onset took place after admission to hospital. Patients with a subarachnoid hemorrhage (ICD‐10: I60.0‐I60.9) or extracranial hemorrhage (ICD‐10: I62.0‐I62.1) were not included in the analyses.

### 2.3 Data collection

Data were collected from the EMS and hospital records, including the hospital diagnosis register. The date of death was obtained from the Swedish National Population Register, together with mortality confirmation.

### 2.4 Statistical analyses

All the data were registered in a database designed for this project. In this statistical analysis, survivors to 1 year were compared with nonsurvivors. Proportions and continuous variables were compared using Fishers exact test and Mann–Whitney U test, respectively. Multivariate analysis was performed using multiple logistic regression. All tests are two‐sided. Owing to the large number of *p* values calculated, *p*‐values below .01 were regarded as statistically significant.

## 3 RESULTS

In all, 1,360 patients with acute stroke were included in the database. Of these, 1,028 patients (76%) were transported by the EMS and are included in this study. Of these 1,028 patients, 360 (35%) had died 1 year after hospital admission. In the following, baseline information before admission to hospital is compared for patients who were still alive 1 year after the stroke and for patients who had died after 1 year.

### 3.1 Baseline characteristics

Patients who died within 1 year after the stroke had a mean age which was 7 years higher than patients who survived (82 years versus 75 years) (Table 1). Furthermore, a history of atrial fibrillation (37% versus 22%), a history of heart failure (17% versus 10%), and a history of prior stroke (36% versus 27%) were more frequent among patients who died. In addition, the presence of cancer showed a weak association with increased mortality.

**Table 1:** Baseline characteristics for patients with acute stroke who survived one year after and those who did not

	Dead *n* = 360	Survived *n* = 668	*p* value
Age (mean ± *SD*; years) (0,0)	82.9 ± 8.8	75.4 ± 11.4	<.0001
Gender, %, female (0,0)	53.6	49.7	.24
History of: %			
Diabetes mellitus (0.3, 0,1)	18.9	19.6	.80
Hypertension (0.3, 0,1)	48.2	52.8	.17
Atrial fibrillation (0.3, 0,3)	37.3	22.2	<.0001
Heart failure (0.3, 0,4)	17.3	9.5	<.0001
Prior myocardial infarction (0.6, 0,4)	17.6	17.6	1.0
Angina pectoris (0.6, 0,4)	8.4	6.9	.45
Prior stroke (0.3, 0,1)	36.5	26.8	.002
Intermittent claudication (0.3, 0,3)	3.3	2.0	.20
Cancer (0.6, 0,3)	17.0	12.0	.04

Numbers in parentheses are the percentages of patients with missing information among those dead and surviving.

### 3.2 Clinical findings and level of priority

Patients who died differed from survivors as follows. They more frequently showed weakness in an arm or a leg, they more often had an oxygen saturation below 90%, they more often had a heart rate over 100, they were given a higher priority by the EMS clinician, and they had a lower level of consciousness. In addition, numbness was more seldom present as a symptom. The level of blood pressure was not associated with the risk of death (Table 2).

**Table 2:** Clinical findings in the ambulance and priority given by the EMS

	Dead *n* = 360	Survived *n* = 668	*p* value
Neurological symptoms			
Weakness arm (16, 5)	55.6	42.5	<.0001
Weakness leg (16, 5)	50.3	38.6	<.0001
Facial droop (20, 9)	31.0	26.5	.18
Numbness (29, 12)	7.1	14.3	.003
Speech disturbance (21, 7)	54.0	47.1	.054
Cardiorespiratory findings			
Oxygen saturation <90% (7, 5)			
Yes	14.9	1.9	<.0001
Systolic blood pressure (mmHg) (5, 4)			
<100	2.4	0.9	.09
>140	71.8	73.1	.71
>200	10.9	11.5	.83
Diastolic blood pressure (mmHg) (8, 8)			
>90	45.4	45.6	1.0
>120	6.4	6.2	.89
Heart rate (beats/min) (5, 3)			
>100	17.0	9.9	.002
>50	1.5	1.1	.76
Priority given by EMS clinician (0.8, 1)			
1	40.9	29.6	<.0001
2	48.2	52.3	
3	10.9	18.0	
Consciousness (RLS‐85) (19, 16)			
1	55.5	88.8	<.0001
2	19.0	8.2	
3	10.3	1.6	
≥4	15.2	1.4	

EMS, emergency medicine services; RLS‐85, Reaction Level Scale.

Numbers in parentheses are the percentages of patients with missing information among those dead and surviving.

### 3.3 Independent predictors of risk of death

In a multivariate analysis, the following appeared as independent predictors of death during the following year: increasing age, a history of heart failure, oxygen saturation below 90%, and a decreased level of consciousness (Table 3). Two further factors tended to be associated with the risk of death: a heart rate above 100 beats/min (*p* = .033) and weakness in a leg (*p* = .039).

**Table 3:** Prehospital factors associated with risk of death during one year of follow‐up

	Univariate		Multivariate		*p* value
	OR	95% Cl	OR	95% Cl	
Age (years)	1.08	1.06–1.10	1.07	1.05–1.09	<.0001
Previous history					
Heart failure	2.00	1.37–2.91	2.08	1.26–3.42	.004
Atrial fibrillation	2.00	1.51–2.64	1.02	0.68–1.54	n.s.
Stroke	1.57	1.19–2.06	1.27	0.86–1.86	n.s
Cardiorespiratory findings					
Heart rate >100 beats/min	1.87	1.27–2.74	1.88	1.04–3.34	.033
Oxygen saturation <90%	9.07	4.92–18.10	8.05	3.33–22.64	<.0001
Neurological symptoms					
Degree of consciousness (RLS‐85 four levels)	2.93	2.38–3.68	2.19	1.61–3.03	<.0001
Weakness arm	1.69	1.28–2.23	1.10	0.69–1.76	n.s.
Weakness leg	1.62	1.22–2.13	1.47	1.02–2.12	.039

RLS‐85: Reaction Level Scale.

## 4 DISCUSSION

In this study, the overall mortality rate during the first year after the onset of stroke was 35%. This figure could be regarded as relatively high. The case fatality rate after stroke has decreased in recent years and previous surveys have shown slightly lower mortality (Gulliford, Charlton, Rudd, Wolfe, & Toschke, 2010). However, one of the strongest risk factors for death was age and the mean age in the present study population was 76 years, which is higher than in many other studies (Gulliford et al., 2010).

We were able to identify four factors, which were strongly associated with the risk of death during 1 year after the onset of stroke, and another two factors which tended to show an associated with the risk of death. These six factors reflect different pathophysiological aspects of the acute stroke disease.

The first and the most unsurprising of these factors was age. For each added year, the adjusted risk of death increased by about seven per cent. A number of previous studies have documented that, with increasing age, the risk of death increases in stroke (Andersen, Andersen, & Olsen, 2011), as well as in many other cardiovascular diseases. The mechanisms behind these findings are multifactorial. Most probably, the most important is the fact that, although a number of diseases were considered, the total spectrum of increasing comorbidity could not be included in our multivariate analysis. Hidden confounders influencing the risk of death were therefore not adjusted for. Theoretical examples are age‐dependent structural changes, such as more severe atherosclerosis, in central organs such as the brain and the heart (which were not manifested as a defined disease but were still important for the outcome).

The second factor was a history of heart failure. The adjusted risk of death in patients who had a history of heart failure was about twice as high compared with those who did not have heart failure. Patients with heart failure run a risk of stroke due to cardiac emboli, especially if there is an association with atrial fibrillation (Lip, Nieuwlaat, Pisters, Lane, & Crijns, 2010). It is well known that cardiac emboli cause a larger infarction with a poorer prognosis compared with no emboli (Stanko & Levine, 2003). Another explanation might be that the heart failure itself increases the risk of cardiac death, due to a successive deterioration in heart function and sudden unexpected death due to a cardiac arrhythmia. The 1‐year mortality among patients with heart failure is improving but is still as high as almost 20% (Sartipy, Dahlström, Edner, & Lund, 2014). The heart failure also reduces the patients’ physical activity and might thereby lead to less successful rehabilitation.

The third factor was a low oxygen saturation. The adjusted risk of death in patients who had an oxygen saturation of less than 90% was about eight times higher compared with patients who did not have an oxygen desaturation of this kind. An oxygen saturation of <90% is uncommon in the prehospital setting of stroke, but, on the other hand, it appears to be the strongest determinant of an adverse outcome. The two most plausible mechanisms behind oxygen desaturation are either respiratory dysfunction or cardiac dysfunction. As we adjusted for a history of heart failure, the possibility of respiratory dysfunction is more likely. Unfortunately, we do not know whether or not these patients had a previous history of a respiratory dysfunction. The association between respiratory dysfunction and stroke is less well described in the literature. It can be assumed that the stroke needs to be very extensive in order to affect the respiratory center. On the other hand, a history of chronic obstructive pulmonary disease has been shown to be associated with an increased risk of sudden death (van den Berg, Stricker, Brusselle, & Lahousse, 2016). We simply do not know whether the oxygen desaturation was a complication of the stroke or whether it was a clinical observation which was independent of the stroke.

The fourth factor was a decreased level of consciousness. For each decrease on a four‐level scale of consciousness, the adjusted risk of death increased about twice. The hypothesis that more extensive strokes may reduce the level of consciousness is not a controversial issue and it has previously been reported that a stroke with more severe symptoms has a higher mortality rate (Adams et al., 1999). Our finding was therefore not unexpected. However, we do not know anything about the eventual fluctuation in the level of consciousness before death.

An elevated heart rate also tended to be associated with the risk of death during follow‐up (*p* = .033). The elevation of heart rate as a risk factor for premature death has been the subject of debate for decades (Boudoulas, Borer, & Boudoulas, 2015). Relative overactivity of the sympathetic nervous system in relation to the parasympathetic nervous system is the most common explanation for an elevated heart rate. The association between the severity of stroke and the elevation of heart rate is more speculative. However, a disturbance in the autonomous nervous system in association with stroke is well known. The hypothesis that can be raised from this study is that, even in stroke, as in heart disease (Thang, Karlson, Sundström, Karlsson, & Herlitz, 2015), the early overactivity of the sympathetic nervous system is associated with an increased risk of death.

The second factor that that showed a borderline significance with the risk of death was the symptom of weakness in a leg. This may, of course, also reflect a more severe stroke, but it might also increase the risk of complications, such as pulmonary emboli (Kamphuisen, Agnelli, & Sedbastianelli, 2005). Unfortunately, data on stroke severity such as the NIHSS‐scale (17) were not available. Instead, we analyzed different stroke symptoms separately.

Among the six factors that were or tended to be independently associated with an increased risk of death in the early phase of stroke, only two were appropriate for early intervention, that is low oxygen saturation and an elevated heart rate. We do not know anything about the possible effects of intensive treatment with oxygen or a pharmaceutical reduction in heart rate in the early phase of stroke. Recent experiences from cardiology suggest that early treatment with oxygen in suspected acute myocardial infarction does not reduce the risk of death during 1 year of follow‐up (Hofmann et al., 2017).

Previous research has shown that both very high and very low blood pressure are associated with poor outcome, linked to both impaired functional outcome and increased mortality (Vemmos et al., 2004). In the present study, no association was found between blood pressure and one‐year mortality. The reason for these diverging results is not obvious and needs to be further studied.

### 4.1 Strengths and limitations

The hospital admissions took place in 2011 and the one‐year follow‐up was completed in 2012. This could be regarded as both a strength and a limitation. As, at the time of the study, thrombectomy was rare and fewer patients than today received thrombolysis, the true biological association between prehospital factors and outcome could more accurately assessed at that time. If we were, however, interested in the way these interventions influence the association between prehospital factors and outcome, a more appropriate answer would have been obtained today.

This was a retrospective analysis of register data. Data were missing for most of the neurological variables. This might be due to trouble examining the patient or lack of time and is unfortunately common in retrospective studies. Further, we based our study on patients with a confirmed diagnosis of stroke on the basis of ICD codes that were created after hospital admission. The physicians’ diagnoses were, however, never validated. Furthermore, the EMS clinician did not suspect a stroke in the prehospital setting in all these cases. In fact, in some additional cases, there was a prehospital suspicion of stroke which was never confirmed in the hospital database.

## 5 CLINICAL IMPLICATIONS

Among patients who use the EMS due to stroke, some risk indicators of an adverse outcome can already be seen at the scene. Abnormal findings in any of the three vital parameters, degree of consciousness, oxygen saturation, and heart rate, are critical. However, the level of blood pressure appears to be less critical. Furthermore, if the patient has a history of heart failure and a higher age, this also indicates an increased risk.

In the future, risk scores may already be created in the prehospital setting for patients with various clinical conditions. This information may form the basis of the creation of a score of this kind.

## 6 CONCLUSION

Among patients with a confirmed diagnosis of stroke, several factors that were associated with the risk of death during the following year could already be identified before arrival at hospital. They were reflected in the patients’ age, previous clinical history, respiratory function, and the function of the central nervous system.

## CONFLICT OF INTERESTS

None declared.
